# Triple jeopardy: transscaphoid, transcapitate, transtriquetral, perilunate fracture dislocation

**DOI:** 10.1007/s10195-012-0195-x

**Published:** 2012-04-04

**Authors:** Bhavuk Garg, Tarun Goyal, Prakash P. Kotwal

**Affiliations:** 1 Department of Orthopaedics, All India Institute of Medical Sciences, New Delhi, India; 2 287 Heaton Road, Newcastle Upon Tyne, NE6 5QD UK

**Keywords:** Perilunate dislocation, Transscaphoid, Transtriquetral, Transcapitate, Carpal injuries

## Abstract

Carpal injuries are frequently underdiagnosed and underreported injuries of the hand. Scapholunate perilunate dissociation is the most common perilunate instability pattern seen in clinical practice. Transscaphoid, transtriquetral, transcapitate dislocation with a volar intercalated segment instability pattern is a very rare pattern of carpal injury. We describe a case with this unique pattern of injury, explaining its mechanism and treatment. Good outcome can be achieved in these injuries following open reduction and internal fixation with ligamentous repair.

## Introduction

Carpal injuries are frequently underdiagnosed and underreported injuries of the hand. Scapholunate perilunate dissociation is the most common perilunate instability pattern seen in clinical practice [1, 2]. Perilunate dislocations may be due to ligamentous injuries (following the lesser arc) or osseoligamentous disruption combining fracture of one or more carpal bones with ligament injuries (the greater arc route). Various greater arc injury patterns have been identified, among which transscaphoid is the commonest, followed by transscaphoid, transcapitate, perilunate fracture dislocation [3, 4]. Other uncommon patterns include transscaphoid, transcapitate, transhamate fracture dislocation, transscaphoid, transcapitate, transtriquetral, perilunate fracture dislocation, and transscaphoid, transtriquetral, perilunate fracture dislocation [5–8].

Transscaphoid, transcapitate, transtriquetral dislocation is a very rare pattern of carpal injury, with only two cases reported in the literature [5, 9]. A unique feature of this injury is its volar intercalated segment instability (VISI) pattern. Below, we describe a case with this unique pattern of injury, explaining its mechanism and treatment.

## Case report

**Fig. 1:**
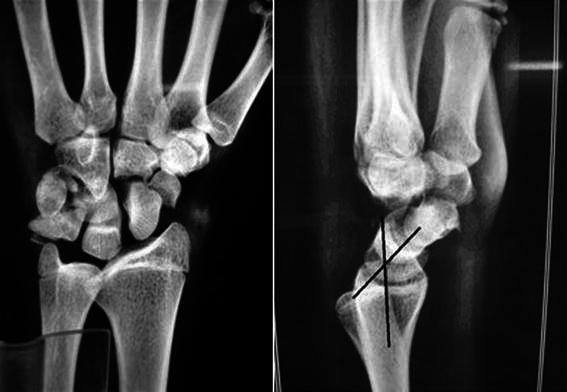
Preoperative radiograph showing fracture of the scaphoid, capitate, and triquetrum, with perilunate dislocation. The radiolunate angle is marked in the figure

A 16-year-old girl presented to our outpatient department with injury to her nondominant left wrist. She was a college student and had a fall from bike with the volar aspect of her hand coming to rest against the ground and her wrist going into hyperextension. There was swelling over her wrist and hand, but no visible open injury. Distal neurovascular status was intact. Posteroanterior and lateral views of the hand showed a displaced fracture of the scaphoid and capitate and a comminuted fracture of the triquetrum. On lateral projection, the lunate was tilted ventrally (VISI); the radiolunate angle was about 55°. The scapholunate angle was about 15° (Fig. 1). Thus, the line of dissociation of the carpal bones followed the greater arc injury pattern, with a transscaphoid, transcapitate, and lunotriquetral disruption.

**Fig. 2:**
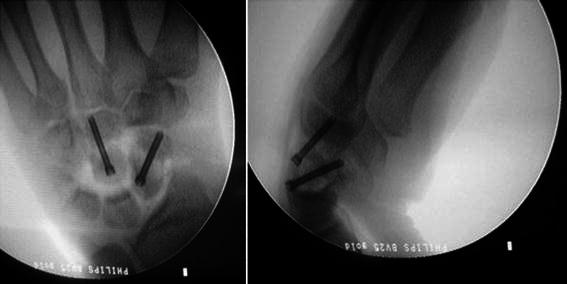
Intraoperative radiograph showing reduction and screw fixation of the scaphoid and capitate. The capitate appears to be aligned with the lunate on the lateral view

Surgery was carried out 5 days following injury under general anesthesia. Scaphoid and capitate were reduced using a dorsal approach and fixed using Herbert–Whipple screws (Fig. 2). The capitate fracture ends were found to be rotated by about 180°. Reconstruction of the scaphocapitate ligament was done using suture anchors. The scapholunate ligament was found to be intact at the time of surgery. Primary repair of the lunotriquetral ligament was carried out and buttressed with the volar capsule. Examination under anesthesia revealed instability of the distal radioulnar joint, for which fixation was done using a K-wire. This K-wire was removed at 3 weeks. Carpal alignment was found to be satisfactory and stable on intraoperative radiographs.

**Fig. 3:**
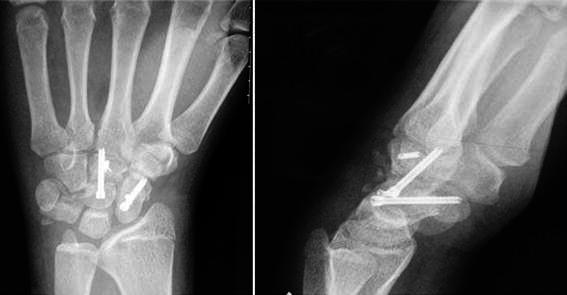
Radiographs at follow-up showing optimally reduced fractures of the scaphoid and capitate and reduction of the perilunate dislocation

Postoperative radiographs revealed anatomical reduction of the scaphoid and capitate, with maintenance of the carpal bone alignment (Fig. 3). A short arm splint was used for 2 weeks, after which active and passive mobilization was started. At 6 months, the fractures had healed and the range of motion at the wrist was about 80° of dorsiflexion and 85° of plantarflexion. There was no pain during wrist movements. There was no restriction of supination or pronation, and no ulnar or radial deviation compared to the other side. Informed consent was obtained from the patient and from her parents for this report.

## Discussion

Perilunate fracture dislocations can have a complex pattern of ligament and bony injuries. Transscaphoid perilunate fracture dislocation is the most common pattern of perilunate injury. Fenton [3] reported a case of simultaneous fracture of the capitate and the scaphoid (naviculocapitate syndrome). Several cases of transscaphoid, transcapitate, perilunate dislocation have been reported in the literature, but transscaphoid, transcapitate, transtriquetral, perilunate dislocations are extremely rare.

The direction of the forces that acted across the carpal bones at the time of injury can be postulated based on the radiological findings. The injury resulted from the hyperextension forces, with the ligaments on the ulnar side giving way first, resulting in the VISI pattern. This contrasts with the more common mechanism of perilunate injury (Mayfield’s classification), where the forces cause disruption of the ligaments from the radial side, resulting in a dorsal intercalated segment instability (DISI) pattern [10]. This reverse Mayfield pattern then resulted in the transcapitate and transscaphoid disruptions. This would happen with an axial load on a dorsiflexed hand, with the hypothenar part bearing the deceleration forces. After the ulnar aspect of the hand impacts the ground, there is forced dorsiflexion and intercarpal pronation, followed by the radial deviation. This causes lunotriquetral dissociation in the form of a lunotriquetral ligament tear or avulsion or triquetrum fracture. Next, the scaphoid comes into contact with the dorsal lip of the distal radial articular surface, resulting in a scaphoid fracture. Thus, ulnar stablizers of the lunate are lost, causing it to tilt volarly. This is a rare mechanism of injury. Not only are ulnar injuries to the carpal bones rare, but they are also poorly understood and diagnosed.

Another point of interest in this patient was the unusual shape of the distal radial articular surface. A certain degree of hypoplasia of the ulnar part of the distal radial physis is evident on the radiographs. Though negative ulnar variance was maintained, there was increased slope of the distal radial articular surface. This is consistant with a milder form of Madelung’s deformity [11]. There would be some associated stretching and weakening of the distal radioulnar joint. Also, the lunate will be proximally and ulnarly migrated because of the deformity, leading to abnormal stresses across the lunatotriquetral joint. This would have led to the intrinsic weakening of the ligaments around these joints, prompting these ligaments to give way at the first instance.

The dorsal approach is the most commonly used approach for the reduction and fixation of perilunate injuries. It provides good exposure of the intercarpal relations, facilitating an assessment of adequate reduction. The scapholunate ligament is generally preserved in a transscaphoid injury. The scaphocapitate ligament and lunotriquetral ligaments were found to be torn at the time of surgery and were repaired. Repairing the ligaments is important, as it can prevent a loss of reduction. This patient received adequate reduction intraoperatively, which was maintained during the follow-up.

Thus, transscaphoid, transcapitate, transtriquetral, perilunate dislocations are extremely rare injuries, and are best treated using open reduction and internal fixation with ligamentous repair. A good outcome can be achieved for these injuries following fixation.
